# Spatial scaling of soil microbial co‐occurrence networks in a fragmented landscape

**DOI:** 10.1002/mlf2.12073

**Published:** 2023-06-26

**Authors:** Pandeng Wang, Shao‐Peng Li, Xian Yang, Xingfeng Si, Wen‐Jun Li, Wensheng Shu, Lin Jiang

**Affiliations:** ^1^ State Key Laboratory of Biocontrol, School of Ecology & School of Life Sciences Sun Yat‐Sen University Guangzhou China; ^2^ School of Biological Sciences Georgia Institute of Technology Atlanta Georgia USA; ^3^ Zhejiang Tiantong Forest Ecosystem National Observation and Research Station, School of Ecological and Environmental Sciences East China Normal University Shanghai China; ^4^ Institute of Eco‐Chongming (IEC) Shanghai China; ^5^ School of Life Sciences South China Normal University Guangzhou China

## Abstract

Habitat loss has been a primary threat to biodiversity. However, species do not function in isolation but often associate with each other and form complex networks. Thus, revealing how the network complexity and stability scale with habitat area will give us more insights into the effects of habitat loss on ecosystems. In this study, we explored the relationships between the island area and the network complexity and stability of soil microbes. We found that the complexity and stability of soil microbial co‐occurrence networks scale positively with island area, indicating that habitat loss will potentially simplify and destabilize soil microbial networks.

The positive species‐area relationship (SAR), which describes how species diversity increases with habitat area, is considered as one of the few general laws in ecology^1^. The positive SAR implies a negative effect of habitat loss on species diversity, a prediction borne out in many studies conducted across the world^2^, ^3^. However, beyond diversity, species often associate with each other and form complex ecological networks in ecosystems. Therefore, revealing the network‐area relationships (NARs) is necessary for gaining deeper insights into the potential effects of habitat loss on the organization of impacted communities^4^. Nevertheless, empirical studies of NARs are remarkably rare and restricted to macroscopic food webs and pollination networks^5^, ^6^. We know virtually nothing about NARs in microbial communities, even though microbes play a crucial role in various biogeochemical processes and supporting higher‐trophic‐level organisms. In recent years, there has been widespread utilization of microbial co‐occurrence network analyses^7^, ^8^, ^9^, ^10^, which have provided an important dimension to characterize community organization beyond species diversity and community composition^11^, ^12^. For instance, the complexity of microbial co‐occurrence networks is known to be related to community stability^13^, ecosystem functioning^14^, and climate warming^15^. However, how the complexity of microbial co‐occurrence networks scales with spatial extent remains largely unknown. One important, but unanswered, question is whether habitat reduction would have a general negative effect on the complexity of microbial co‐occurrence networks (i.e., positive NARs), similar to its negative effect on microbial diversity^16^.

NARs may arise from various mechanisms^4^. For instance, the different shapes of SARs across trophic levels could cause the spatial scaling of food web structure, which has been demonstrated by both theoretical and empirical studies^4^, ^5^, ^6^. Following similar logic, we might expect that increased diversity in larger areas, as predicted by the SARs, would increase the likelihood of more taxa interacting with each other, potentially resulting in increased complexity of microbial networks. Furthermore, larger islands, when harboring higher‐quality habitats than smaller islands due to reduced edge effects and improved soil conditions^17^, could support more microbial individuals per unit of area, which would translate into increased encounter probability of different microbial taxa. Thus, higher habitat quality on larger islands could result in increased microbial network complexity, and in turn, positive network complexity‐area relationships. However, how microbial diversity and habitat quality (e.g., soil properties) contribute to the NARs has not been explored empirically.

The complexity of an ecological system has long been thought to influence its stability^18^, ^19^. Recent theoretical studies^20^ have shown that network complexity (e.g., network size, connectivity/links, connectance, lineage density/average degree) and architecture (e.g., modularity, nestedness) strongly affect the stability of food webs^21^, mutualistic networks^22^, and antagonistic networks^23^. For example, high connectance has been found to promote the stability of mutualistic networks but decrease the stability of food webs^22^. The effects of habitat loss and fragmentation on the complexity and stability of food webs and mutualistic networks have also been reported^20^, ^24^, ^25^, ^26^, ^27^, ^28^. However, we still know remarkably little about how habitat loss influences the structure and stability of soil microbial networks. Soil microbes (e.g., bacteria and fungi) are essential components of terrestrial ecosystems, and their stability is important for maintaining stable terrestrial ecosystem functions in the face of ongoing changes. Thus, revealing how the stability of soil microbial networks scales with the area will be necessary for comprehensively understanding the ecological consequences of habitat loss.

In this study, we explored the spatial scaling of microbial networks using soil microbial data collected from 28 islands in the Thousand Island Lake of China. The spatial discreteness and relatively close proximity of these islands offer an excellent opportunity for studying microbial NARs. Here we aimed to answer the following two questions: (1) how do the complexity and stability of soil microbial co‐occurrence networks change with island area? (2) What factors underlie the spatial scaling of complexity and stability of soil microbial co‐occurrence networks in the fragmented landscape?

Utilizing amplicon sequencing, we obtained 10,464,475 bacterial and 4,179,719 fungal high‐quality sequences across all 284 samples, with an average of 36,847 ± 12,129 (mean ± SD) bacterial sequences and 14,717 ± 11,649 fungal sequences per sample. After clustering high‐quality sequences into operational taxonomic units (OTUs) at the 97% similarity level and removing singletons, we obtained 19,938 bacterial OTUs and 10,410 fungal OTUs. After rarefying all samples to equal sequencing depth (bacteria: 8381 reads; fungi: 2720 reads), we obtained an average of 1419 ± 167 bacterial OTUs and 338 ± 51 fungal OTUs per sample. At the island level, the ranges of bacterial OTUs and fungal OTUs were 2599–7707 and 710–2229, respectively (Figure S1).

We constructed soil bacterial and fungal co‐occurrence networks based on the Spearman correlations of OTU abundances (log_1__0_‐transformed) across all 284 samples. For both soil bacterial and fungal networks, we found that network size (*n*), total links (*L*), average degree (average K), and the number of keystone OTUs significantly increased as island area increased (Figure 1), indicating that microbial networks on larger islands included more nodes and more potential interactions between nodes. Meanwhile, the connectance of both bacterial and fungal networks decreased as island area increased (Figure 1), indicating that the interaction densities in soil microbial networks were smaller on larger islands. Relative modularity (RM), which measures the degree to which a network is compartmentalized into different modules, significantly increased as island area increased in soil bacterial networks but showed a nonsignificant relationship with island area in soil fungal networks (Figure 1). Additionally, to examine whether these findings are affected by indirect associations, we used the iDIRECT (Inference of Direct and Indirect Relationships with Effective Copula‐based Transitivity) framework to disentangle the direct and indirect associations in the soil bacterial and fungal networks. Based on the iDIRECT‐processed networks, we found similar relationships between island area and network properties of soil bacteria and fungi (Figure S2). Together, these results revealed that the structures of both soil bacterial and fungal networks were generally more complex on larger islands.

**Figure 1:**
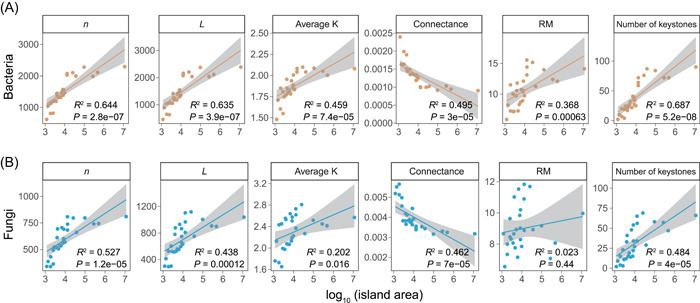
Influences of island area on the structural properties of soil bacterial and fungal networks. (A) Bacteria; (B) Fungi. Network properties include node numbers (*n*), total links (*L*), average node degree (average K), connectance, relative modularity (RM), and the number of keystone OTUs. Island area is log_10_–transformed. Line in each panel was fitted using linear regression with a 95% confidence interval. The adjusted *R* ^2^ and *P* values are shown.

Using random forest analysis, we found that species richness was the most important predictor of network complexity (Figure 2), with *n*, *L*, average K, RM, and the number of keystone OTUs all exhibiting significantly positive relationships with the richness of soil bacteria and fungi (Figures S3 and S4). In addition to species richness, soil quality also had a strong influence on the network complexity of soil bacteria and fungi (Figure 2). Among all the measured soil properties, soil moisture, which exhibited a wide range across the studied islands (ranging from 6.7% to 28.7%), showed strong relationships with network complexities of both soil bacteria (positive: *n*, *L*, average K, RM, number of keystone OTUs; negative: connectance) and fungi (positive: *n*, *L*, average K, number of keystone OTUs) (Figure S3). The random forest analysis also indicated that soil available Ca was an important predictor of bacterial and fungal network complexity (Figure 2), although its correlations with most network properties were not significant (Figure S3). Additionally, total organic carbon (TOC) and total nitrogen (TN) are also positively related with network complexity (*L*, average K, number of keystone OTUs) of soil fungi, but not bacteria (Figure S3). Microbial diversity and measured soil properties accounted for over half of the total variance of network properties, except for the RM of the soil fungal network (Figure 2). Together, our results showed that increased bacterial and fungal richness, as well as increased moisture on larger islands, generally augment the complexity of both bacterial and fungal networks.

**Figure 2:**
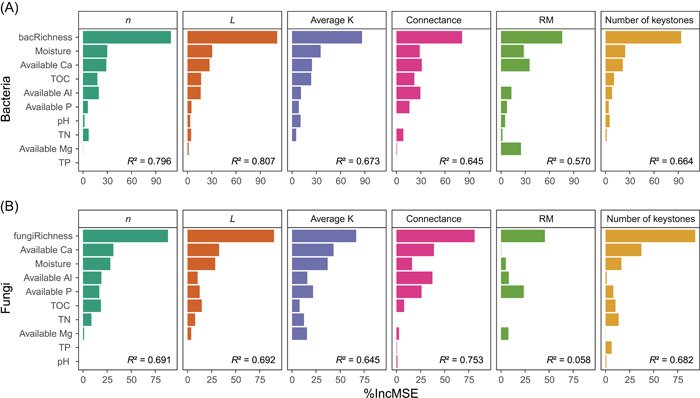
The relative importance of bacterial/fungal richness and different soil properties in driving the variations of soil bacterial and fungal network properties. (A) Bacteria; (B) Fungi. Random forest was used to determine the variable importance. *R* ^2^ (pseudo) represents the total variance that could be explained by environmental factors. The richness of bacteria (bacRichness) and fungi (fungiRichness) was calculated as the total OTU number on each island. Soil properties represent the mean value of all samples on each island. OTU, operational taxonomic units; TN, total N; TOC, total organic carbon; TP, total P.

To assess the stability of microbial networks, we calculated both network robustness and vulnerability. Robustness refers to the resistance of ecological networks to species extinction, whereas vulnerability is an instability measure that assesses the efficiency of the spread of local perturbations across the network (i.e., how fast the effects of environmental changes on impacted nodes transmit to other network components). For both soil bacteria and fungi, network robustness increased, and vulnerability decreased on larger islands (Figure 3A,B), indicating that both soil bacterial and fungal networks exhibited greater stability on larger islands. Similar patterns were found after removing the indirect associations in the microbial networks (Figure S5). As expected, network stability showed significant relationships with network complexity metrics (Figure 3C,D), where the stability of both soil bacterial and fungal networks increased with *n*, *L*, average K, RM, and the number of keystone OTUs, but declined with connectance. Therefore, the increased complexities of both soil bacterial and fungal networks generally promoted network stability on larger islands.

**Figure 3:**
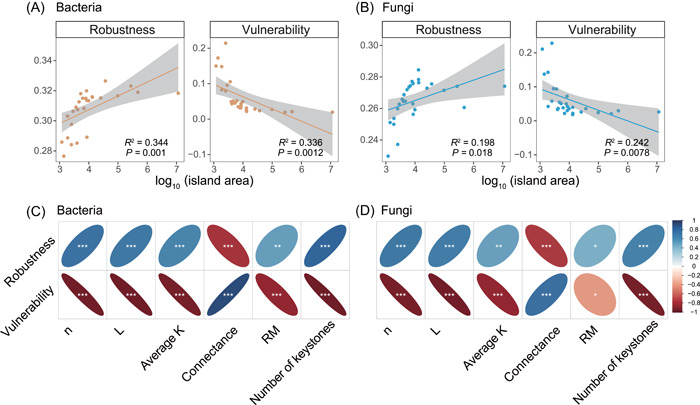
Relationships of network stability with island area and multiple network properties. (A, B) Network robustness was calculated as the remained proportion of taxa after randomly removing 50% of the taxa from each empirical network; network vulnerability was calculated as the maximum node vulnerability in each empirical network. Line in each panel was fitted using linear regression with a 95% confidence interval. The adjusted *R* ^2^ and *P* values are shown. (C, D) Spearman correlations (*P < 0.05, **P < 0.01, ***P < 0.001) of network robustness and vulnerability with different network properties, including *n*, *L*, average K, connectance, RM, and the number of keystone OTUs.

Compared to SARs, our understanding of the NARs is still in its infancy, especially for microorganisms. In this study, we found that the complexities of soil microbial (both bacterial and fungal) networks exhibited positive relationships with island area. Specifically, network size, total links, average degree, relative modularity, and the number of keystone OTUs were all significantly higher on larger islands. These results are consistent with studies of food webs and mutualism networks of macro‐organisms^5^, ^6^, ^26^, ^29^. For instance, Galiana et al. found that the complexity (network size, links, and links per species) of food webs scaled positively with area in both theoretical simulations and empirical observations^4,6^. In our study, the increasing number of keystone taxa was mainly due to the increases in connectors and module hubs on larger islands (Figure S6). However, the network connectance (total links/(network size)^2^) decreased as island area increased (Figure 1), consistent with the previous study of plant–frugivore network^26^ and the theoretical prediction of trophic network^4^, which is attributed to the disproportionate increasing rate of network size and total links along the area. Together, our findings indicate that NARs apply to not only ecological networks of macro‐organisms but also to soil microbes.

Unlike SARs, whose underlying mechanisms are generally well understood^16^, ^30^, we know little about the ecological processes that underlie NARs. Recent theoretical and empirical work has revealed potential mechanisms driving changes in food web structure across spatial scales^4^, ^5^, ^6^. Among those, SARs have been identified as an important driver of NARs.^5^, ^6^ In accordance with these studies, we found that SARs were the main factor that drives NARs of soil bacteria and fungi (Figures 2, S1, S3, and S4). One potential explanation is that higher microbial diversity on larger islands could increase the likelihood of more taxa interacting with each other and in turn, result in increased complexity of microbial networks. In addition to species richness, we found that soil moisture was also a significant predictor of the network complexities of soil bacteria and fungi (Figure S3). Soil moisture, which ranged from 6.7% to 28.7% in our study sites, was generally a limiting factor for microbes on the small islands, because of the low canopy cover, low water holding capacity, and high evapotranspiration on these islands. Previous studies have demonstrated that soil moisture is an important indicator of soil quality in Thousand Island Lake, with higher soil moisture increasing deadwood diversity and termite feeding activity^31^, reducing plant seedling mortality^32^, and promoting coexistence of soil bacteria^16^. Therefore, habitats with higher soil moisture on larger islands would support larger microbial populations^33^ and increase the connectivity of water film, which leads to increased encounter probability of different species and in turn greater network complexity. In line with our findings, microbial diversity and soil moisture were also found to affect the complexity of microbial co‐occurrence networks in both rhizosphere and bulk soils in wheat fields^34^. Additionally, we found that available Ca was also an important predictor of the network complexities of soil bacteria and fungi (Figure 2), possibly due to its strong effects on bacterial and fungal richness^16^, ^35^. Together, our results suggest that the increased bacterial and fungal richness, as well as higher soil quality on larger islands, were responsible for the microbial NARs on our investigated islands.

Both theoretical and empirical studies have shown that the structure of an ecological network can influence its stability^18^, ^20^, ^22^, ^23^. In line with this prediction, we found that network size, total links, average degree, relative modularity, and the number of keystone OTUs all exhibited positive relationships with network stability. Similar relationships between microbial network complexity and stability were also found under climate warming scenarios^15^. In contrast, we found that network connectance exhibited a significantly negative relationship with soil microbial network stability, supporting the notion that lower connectance could mitigate the spread of perturbations (e.g., species extinction) across the network^15^, ^36^. Given the strong relationships between network properties and stability, we found that the stability of the soil microbial network also scaled positively with island area. Therefore, our study indicates that habitat loss may not only affect species diversity but also could reduce the stability of ecological networks by affecting their complexity.

Keystone taxa, which often refer to the nodes (i.e., module hubs, connectors, and network hubs) that occupy key positions in microbial networks, were reported to have important influences on network stability^37^, ^38^. Consistent with these findings, we found that the number of keystone bacterial and fungal taxa exhibited strong positive relationships with network stability (Figure 3). After removing keystone taxa from the bacterial and fungal networks, network robustness significantly decreased on almost all islands (Figure S7), providing further evidence that keystone taxa play an important role in maintaining microbial network stability. As previous studies^37^, ^39^ have shown, the majority of the keystone taxa in our bacterial networks came from *Proteobacteria* and *Acidobacteria*, while keystone taxa in fungal networks are mostly from *Ascomycota* (Figure S8). Among all bacterial keystone taxa, OTUs that belong to families *Acetobacteraceae*, *Xanthomonadaceae*, and *Nitrosomonadaceae*, were identified as module hubs/connectors across most studied islands (Tables S1 and S2), indicating that these taxa may have consistent and important roles in the stability of soil bacterial networks across islands. For fungal communities, OTUs that belong to families *Nectriaceae*, *Mortierella* were identified as the potential core taxa in affecting the stability of soil fungal networks (Tables S3 and S4). These results provide valuable clues for further controlled experiments to identify the keystone taxa that have important roles in maintaining microbial network stability.

It should be noted that, unlike trophic and mutualistic networks of macro‐organisms, the edges between nodes of microbial co‐occurrence networks could not be simply interpreted as biotic interactions^40^. Instead, we should treat the microbial co‐occurrence network as one aspect of the organization of microbial meta‐communities. On the other hand, co‐occurrence network analyses have been widely applied in microbial ecology, providing much useful information beyond diversity^10^. Additionally, it is currently difficult, if not impossible, to reveal the interactions between a large number of microbial taxa in most natural habitats. Note that progress has been made in inferring microbial interactions in natural communities using genome‐scale metabolic modeling^41^, ^42^. It is our hope that similar approaches could be used to explore the spatial scaling of microbial interactions in the future, which would give further insights into the consequences of habitat loss on soil microbial networks.

The positive SAR has been well documented and generally serves as a theoretical foundation for biodiversity conservation. In this study, we further demonstrated that the complexities and stability of both bacterial and fungal networks scaled positively with island area, suggesting that habitat loss could simplify and destabilize soil microbial networks. Additionally, our findings also indicate that the properties of soil microbial co‐occurrence networks are dependent on the scale of sampling, highlighting the need to consider the sampling area when comparing network properties to generate meaningful conclusions. Given the critical roles of soil microbes in terrestrial ecosystems, it is essential to pay more attention to the effects of habitat loss on soil microbial networks for comprehensively understanding the ecological consequences of habitat loss in the future.

## AUTHOR CONTRIBUTIONS

**Pandeng Wang**: Conceptualization (equal); data curation (equal); formal analysis (lead); methodology (lead); visualization (lead); writing—original draft (lead); writing—review and editing (equal). **Shao‐Peng Li**: Conceptualization (equal); data curation (equal); funding acquisition (equal); project administration (equal); supervision (equal); writing—review and editing (equal). **Xian Yang**: Data curation (supporting); writing—review and editing (supporting). **Xingfeng Si**: Writing—review and editing (supporting). **Wen‐Jun Li**: Supervision (supporting); writing—review and editing (supporting). **Wensheng Shu**: Resources (equal); supervision (supporting); writing—review and editing (supporting). **Lin Jiang**: Funding acquisition (equal); project administration (lead); resources (equal); supervision (equal); writing—review and editing (equal).

## ETHICS STATEMENT

No animals and human were involved in this study.

## CONFLICT OF INTERESTS

The authors declare no conflict of interests.

## Supporting information


**Fig. S1 Changes of bacterial and fungal OTU richness along island area**. (**A, B**) The island level OTU richness was calculated as the total number of OTUs that occurred in samples of that island after rarefying all samples to equal sequence numbers. (**C, D**) After rarefying the OTU table, the OTUs that occurred in less than 12 samples among all samples were filtered before calculating the OTU richness for each island. **Fig. S2 Influences of island area on the structural properties of soil bacterial (A) and fungal iDIRECT‐processed networks (B)**. iDIRECT (Inference of Direct and Indirect Relationships with Effective Copula‐based Transitivity) was used to remove indirect associations in the microbial networks. Network properties include node numbers (*n*), total links (*L*), average node degree (Average K), connectance, relative modularity (RM), and the number of keystone nodes. Island area is log_10_ transformed. Line in each panel was fitted using linear regression with a 95% confidence interval. The adjusted R^2^ and *P* values are shown. **Fig. S3 Spearman correlations between environmental factors and network properties of soil bacteria (A) and fungi (B)**. The richness of bacteria (bacRichness) and fungi (fungiRichness) was calculated as the total OTU number on each island. Other environmental factors represent the mean value of all samples on each island. The number inside each cell is the corresponding correlation coefficient. Non‐significant correlations (*P* > 0.05) are marked in grey. **Fig. S4 Influences of island‐level bacterial and fungal richness on the structural properties of soil bacterial (A) and fungal networks (B), respectively**. Network properties include node numbers (*n*), total links (*L*), average node degree (Average K), connectance, relative modularity (RM), and the number of keystone nodes. The island‐level richness of bacteria and fungi was calculated as the total OTU number on each island. Line in each panel was fitted using linear regression with a 95% confidence interval. The adjusted R^2^ and *P* values are shown. **Fig. S5 Influences of island area on the stability of soil bacterial (A) and fungal iDIRECT‐processed networks (B)**. iDIRECT (Inference of Direct and Indirect Relationships with Effective Copula‐based Transitivity) was used to remove indirect associations in the microbial networks. Network robustness was calculated as the remained proportion of taxa after randomly removing 50% of the taxa from each empirical network; Network vulnerability was calculated as the maximum node vulnerability in each empirical network. Line in each panel was fitted using linear regression with a 95% confidence interval. The adjusted R^2^ and *P* values are shown. **Fig. S6 Changes of the number of different keystone nodes in soil bacterial (A) and fungal (B) networks along the island area**. Nodes in the network were classified into network hubs (Z_i_ ≥ 2.5, P_i_ ≥ 0.62), module hubs (Z_i_ ≥ 2.5, P_i_ < 0.62), connectors (Z_i_ < 2.5, P_i_ ≥ 0.62), and peripherals (Z_i_ < 2.5, P_i_ < 0.62), according to the within‐module connectivity (Z_i_) and among‐module connectivity (P_i_). **Fig. S7 Robustness (mean ± se) of bacterial and fungal original networks (black point) and keystone‐removed networks (red points)**. Network robustness was calculated as the remained proportion of taxa after randomly (repeat 999 times) removing 50% of the taxa from each original network or keystone‐removed network. Except for the islands labeled by “ns”, the difference between the robustness of keystone‐removed networks and original networks on each island was significant (t.test, *P* < 0.05). Islands are ordered according to their area (from the smallest to largest). **Fig. S8 Changes in the numbers and phylum compositions of potential bacterial and fungal keystone OTUs across islands**. Islands are ordered according to their area (from the smallest to largest). **Fig. S9 The locations of the 29 surveyed islands in Thousand Island Lake, Zhejiang Province, China**. Islands are ordered according to their area (from the smallest to largest). **Fig. S10 Schematic diagram of soil sampling in one island. Fig. S11 The relationship between island area (m** ^**2**^; **log10 ‐transformed) and each soil property (mean ± sd)**. The unit of moisture is %; the unit of all other soil properties is ppm. Grey shadows represent a 95% confidence interval. **Fig. S12 Rarefaction curves of bacterial and fungal communities for each sample**. The vertical red dash lines indicate the minimum sequence numbers that were used for rarefying bacterial (**A**) and fungal (**B**) community of each sample, respectively.

Table S1 Potential keystone bacterial OTUs on each island. **Table S2** The network status of each potential keystone bacterial OTUs on each island. **Table S3** Potential keystone fungal OTUs on each island. **Table S4** The network status of each potential keystone fungal OTUs on each island. **Table S5** Brief information of 28 studied islands.

Supplementary information.

## Data Availability

DNA sequence data are accessible at the NCBI‐SRA under the accession number PRJNA517449. All other data that support the findings of this study have been deposited to figshare (https://doi.org/10.6084/m9.figshare.10728242).
