# Supplementary material for: Spatial scaling of soil microbial co‐occurrence networks in a fragmented landscape
Source: mLife. 2023 Jun 26;2(2):209–15. doi: 10.1002/mlf2.12073 (PMC10989968; doi:10.1002/mlf2.12073)
Supplement: Supplementary file 1 — Fig. S1 Changes of bacterial and fungal OTU richness along island area. (A, B) The island level OTU richness was calculated as the total number of OTUs that occurred in samples of that island after rarefying all samples to equal sequence numbers. (C, D) After rarefying the OTU table, the OTUs that occurred in less than 12 samples among all samples were filtered before calculating the OTU richness for each island. Fig. S2 Influences of island area on the structural properties of soil bacterial (A) and fungal iDIRECT‐processed networks (B). iDIRECT (Inference of Direct and Indirect Relationships with Effective Copula‐based Transitivity) was used to remove indirect associations in the microbial networks. Network properties include node numbers (n), total links (L), average node degree (Average K), connectance, relative modularity (RM), and the number of keystone nodes. Island area is log10 transformed. Line in each panel was fitted using linear regression with a 95% confidence interval. The adjusted R2 and P values are shown. Fig. S3 Spearman correlations between environmental factors and network properties of soil bacteria (A) and fungi (B). The richness of bacteria (bacRichness) and fungi (fungiRichness) was calculated as the total OTU number on each island. Other environmental factors represent the mean value of all samples on each island. The number inside each cell is the corresponding correlation coefficient. Non‐significant correlations (P > 0.05) are marked in grey. Fig. S4 Influences of island‐level bacterial and fungal richness on the structural properties of soil bacterial (A) and fungal networks (B), respectively. Network properties include node numbers (n), total links (L), average node degree (Average K), connectance, relative modularity (RM), and the number of keystone nodes. The island‐level richness of bacteria and fungi was calculated as the total OTU number on each island. Line in each panel was fitted using linear regression with a 95% confidence in [file MLF2-2-209-s002.docx]

**Supplementary figures:**


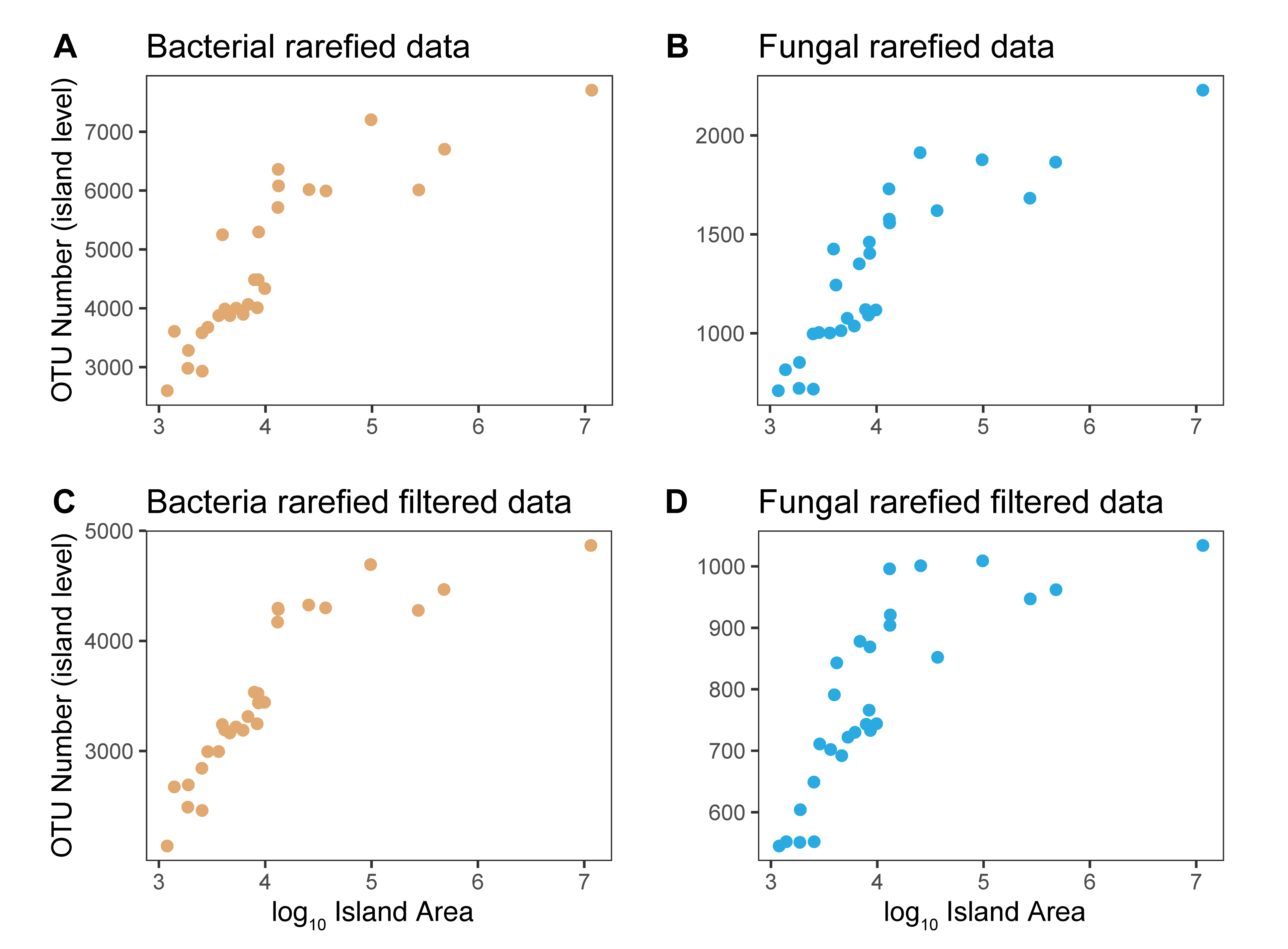


**Fig. S1 Changes of bacterial and fungal OTU richness along island area.** (**A, B**) The island level OTU richness was calculated as the total number of OTUs that occurred in samples of that island after rarefying all samples to equal sequence number. (**C, D**) After rarefying the OTU table, the OTUs that occurred in less than 12 samples among all samples were filtered before calculating the OTU richness for each island.


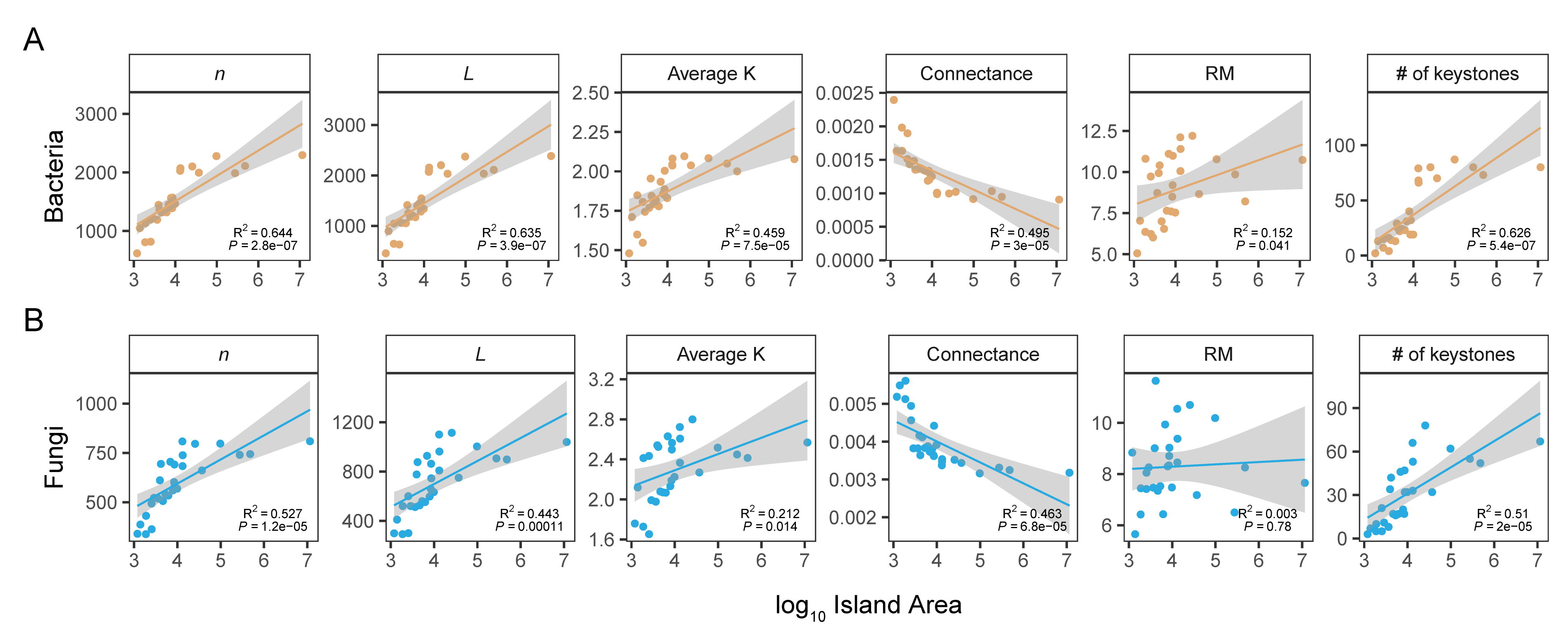


**Fig. S2** **Influences of island area on the** **structure properties of soil bacterial (A) and fungal iDIRECT-processed networks (B)**. iDIRECT (Inference of Direct and Indirect Relationships with Effective Copula-based Transitivity) was used to remove indirect associations in the microbial networks. Network properties include node numbers (*n*), total links (*L*), average node degree (Average K), connectance, relative modularity (RM), and the number of keystone nodes. Island area is log_10_ transformed. Line in each panel was fitted using liner regression with 95% confidence interval. The adjusted R^2^ and *P* values are shown.


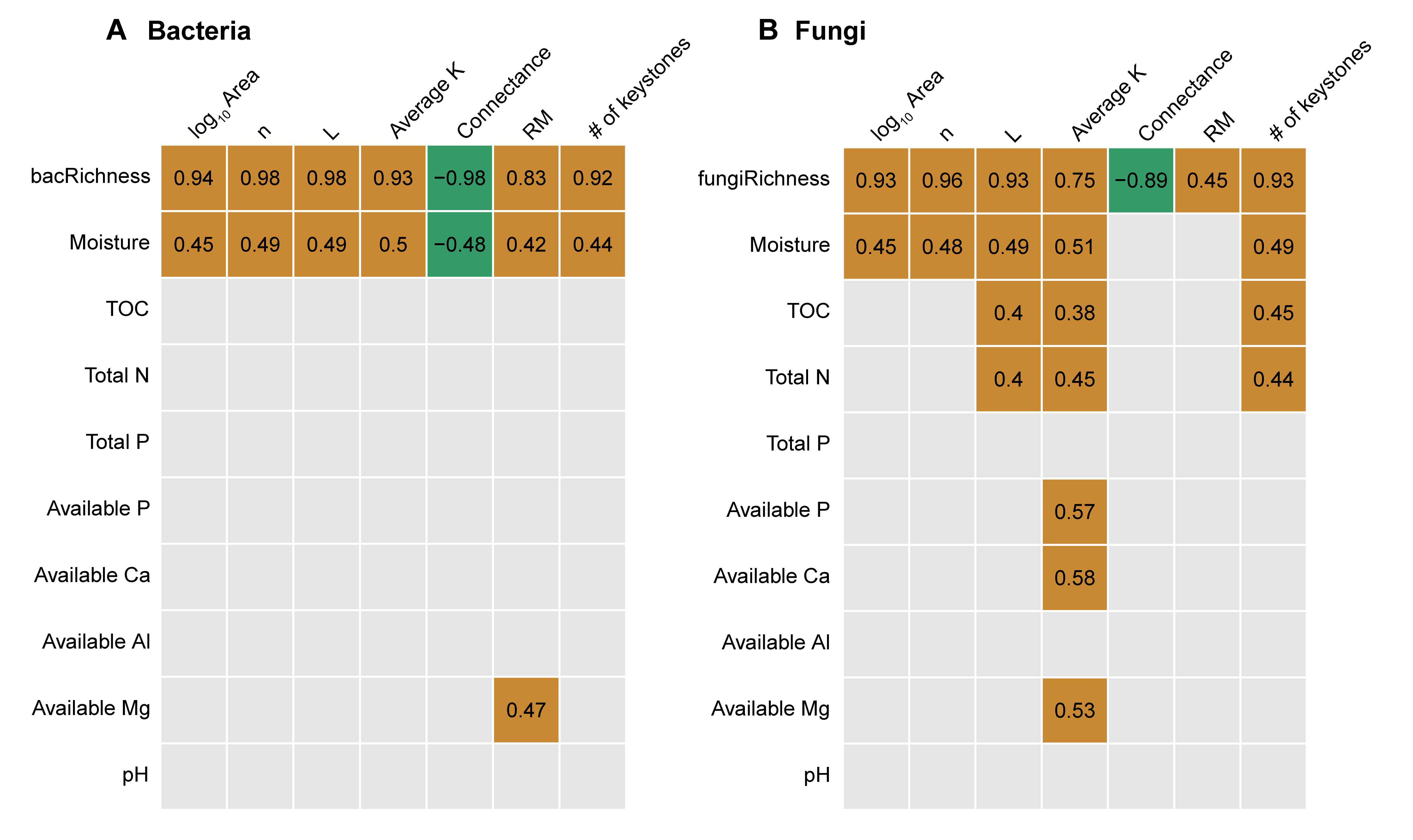


**Fig. S3** **Spearman correlations between environmental factors and network properties of soil bacteria (A) and fungi (B)**. The richness of bacteria (bacRichness) and fungi (fungiRichness) were calculated as the total OTU number on each island. Other environmental factors represent the mean value of all samples on each island. The number inside each cell is the corresponding correlation coefficient. Non-significant correlations (*P* > 0.05) are marked in grey.

**Fig. S4** **Influences of island level bacterial and fungal richness on the structure properties of soil bacterial (A) and fungal networks (B), respectively**. Network properties include node numbers (*n*), total links (*L*), average node degree (Average K), connectance, relative modularity (RM), and the number of keystone nodes. The island level richness of bacteria and fungi were calculated as the total OTU number on each island. Line in each panel was fitted using liner regressin with 95% confidence interval. The adjusted R^2^ and *P* values are shown.


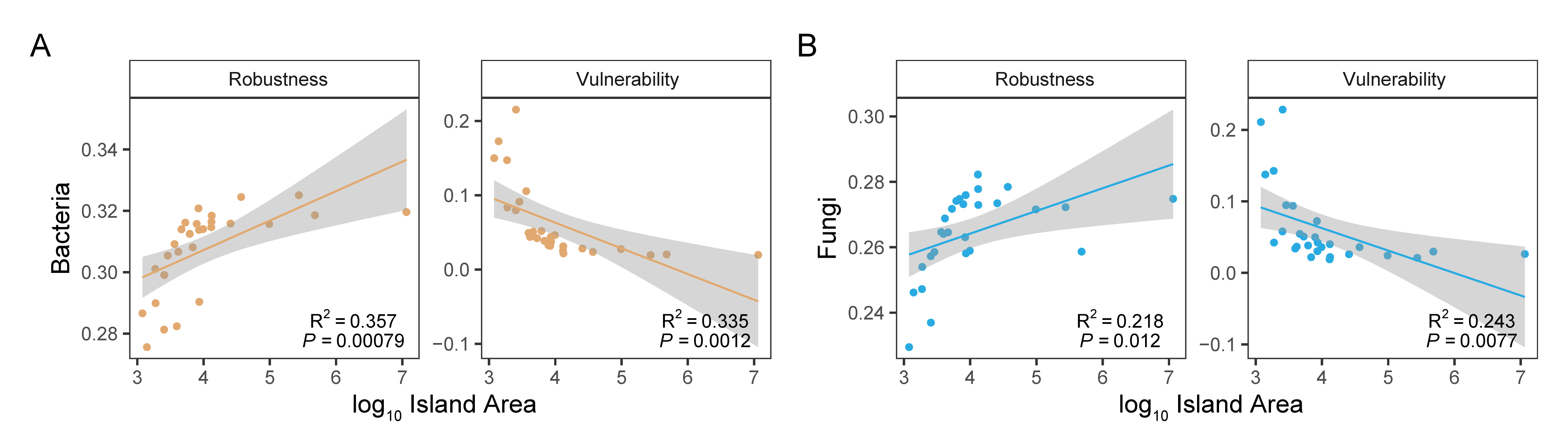


**Fig. S5** **Influences of island area on the stability of soil bacterial (A) and fungal iDIRECT-processed networks (B)**. iDIRECT (Inference of Direct and Indirect Relationships with Effective Copula-based Transitivity) was used to remove indirect associations in the microbial networks. Network robustness was calculated as the remained proportion of taxa after randomly removing 50% of the taxa from each empirical network; Network vulnerability was calculated as the maximum node vulnerability in each empirical network. Line in each panel was fitted using liner regression with 95% confidence interval. The adjusted R^2^ and *P* values are shown.


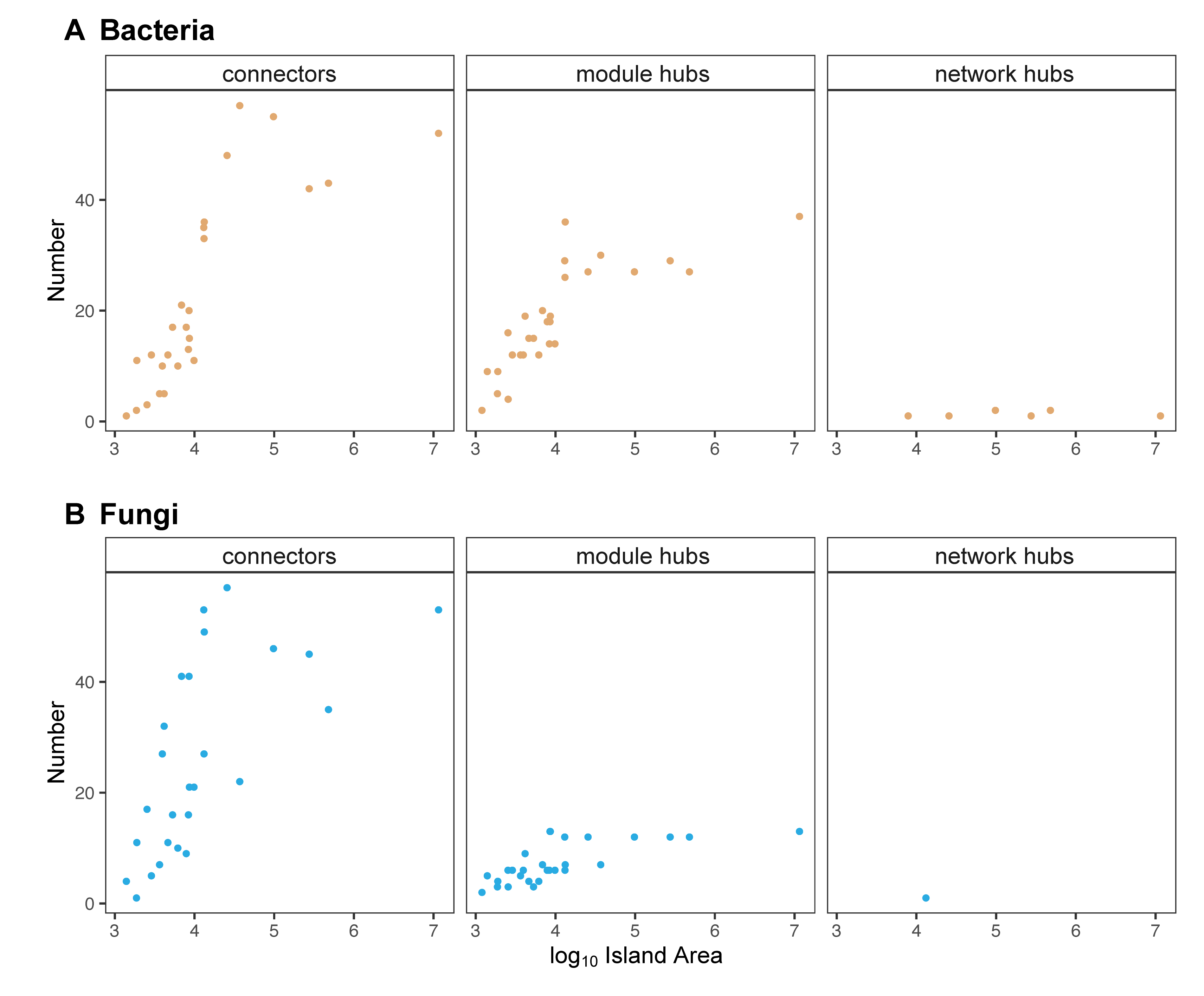


**Fig. S6** **Changes of the number of different keystone nodes in soil bacterial (A) and fungal (B) networks along the island area**. Nodes in the network were classified into network hubs (Z_i_ ≥ 2.5, P_i_ ≥ 0.62), module hubs (Z_i_ ≥ 2.5, P_i_ < 0.62), connectors (Z_i_ < 2.5, P_i_ ≥ 0.62), and peripherals (Z_i_ < 2.5, P_i_ < 0.62), according to the within-module connectivity (Z_i_) and among-module connectivity (P_i_).


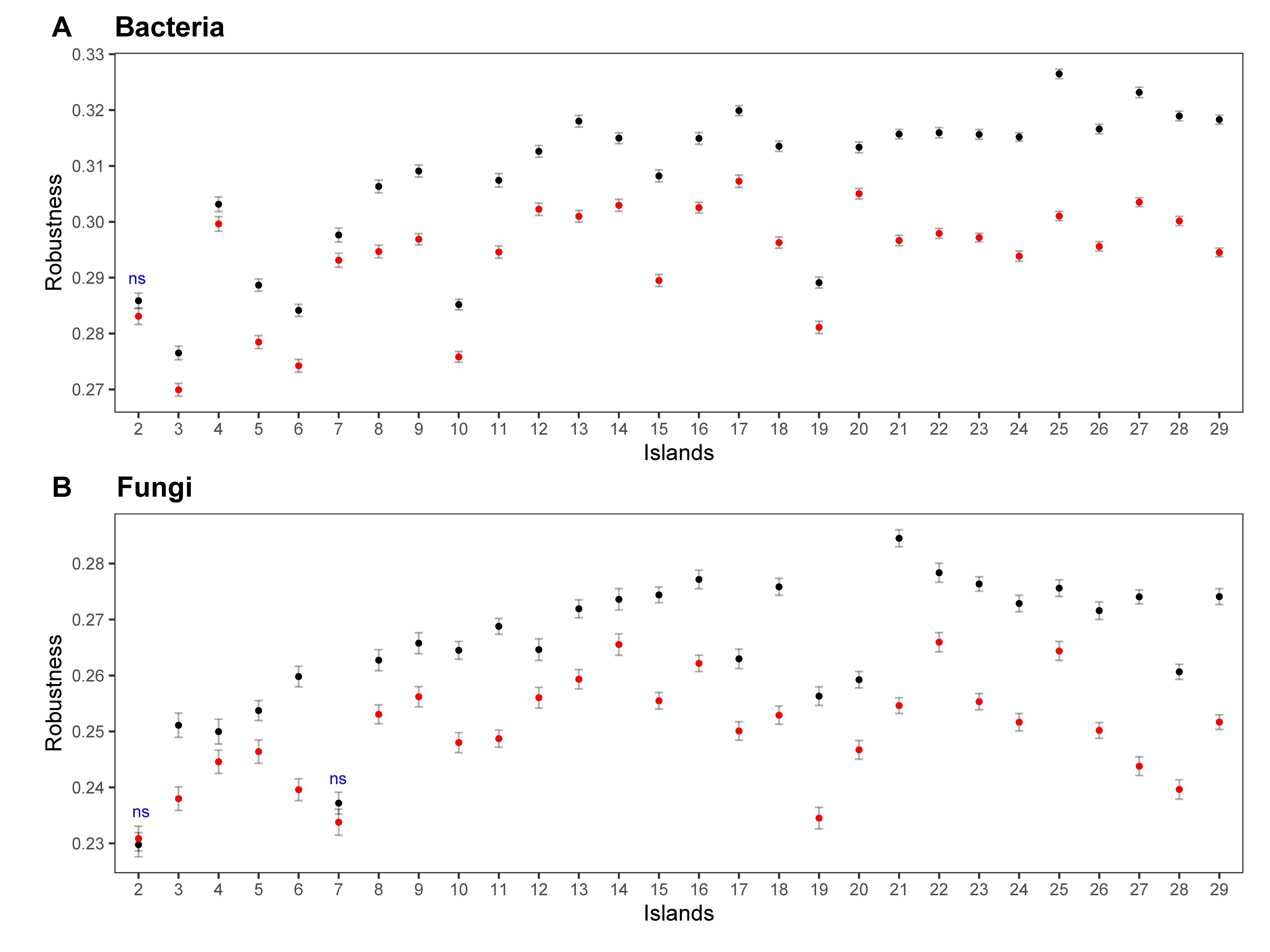


**Fig. S7 Robustness (mean ± se) of bacterial and fungal original networks (black point) and keystone-removed networks (red points).** Network robustness was calculated as the remained proportion of taxa after randomly (repeat 999 times) removing 50% of the taxa from each orginal network or keystone-removed network. Except for the islands labeled by “ns”, the difference between robustness of keystone-removed networks and original networks on each island was significant (t.test, *P* < 0.05). Islands are ordered according to their area (from the smallest to largest).


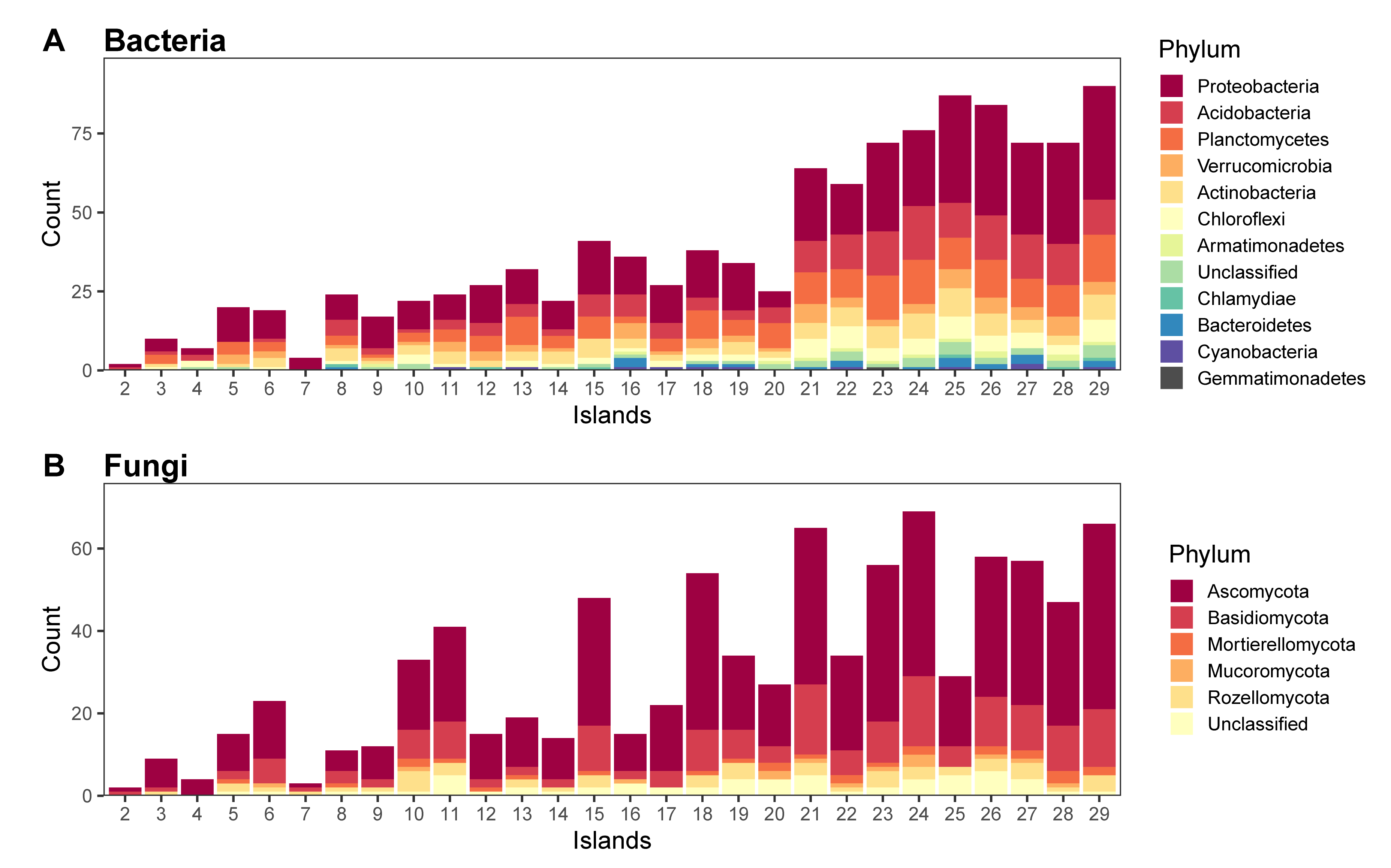


**Fig. S8 Changes of the numbers and phylum compositons of potential bacterial and fungal keystone OTUs across islands.** Islands are ordered according to their area (from the smallest to largest).


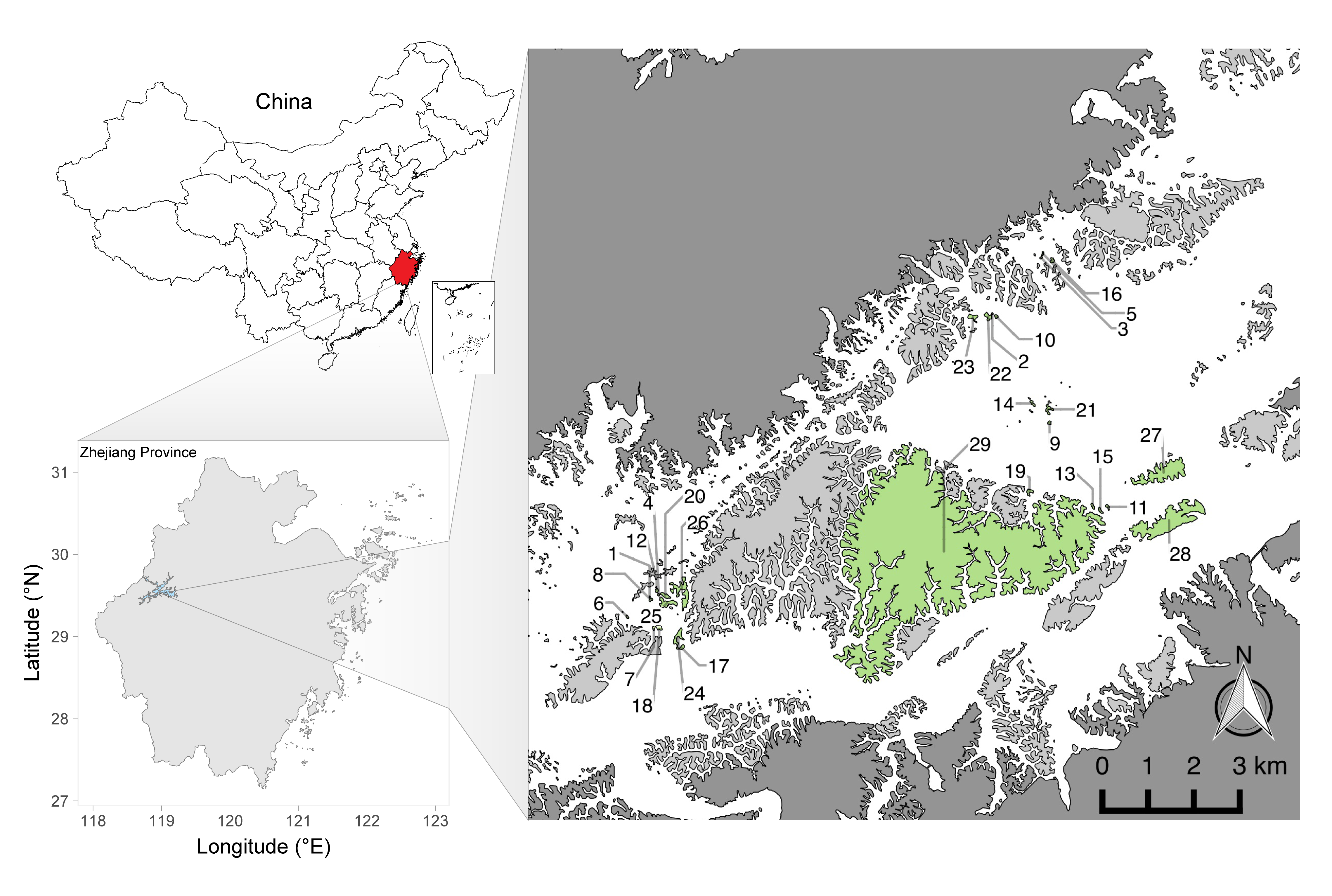


**Fig. S9** **The locations of the 29 surveyed islands in the Thousand Island Lake, Zhejiang Province, China.** Islands are ordered according to their area (from the smallest to largest).


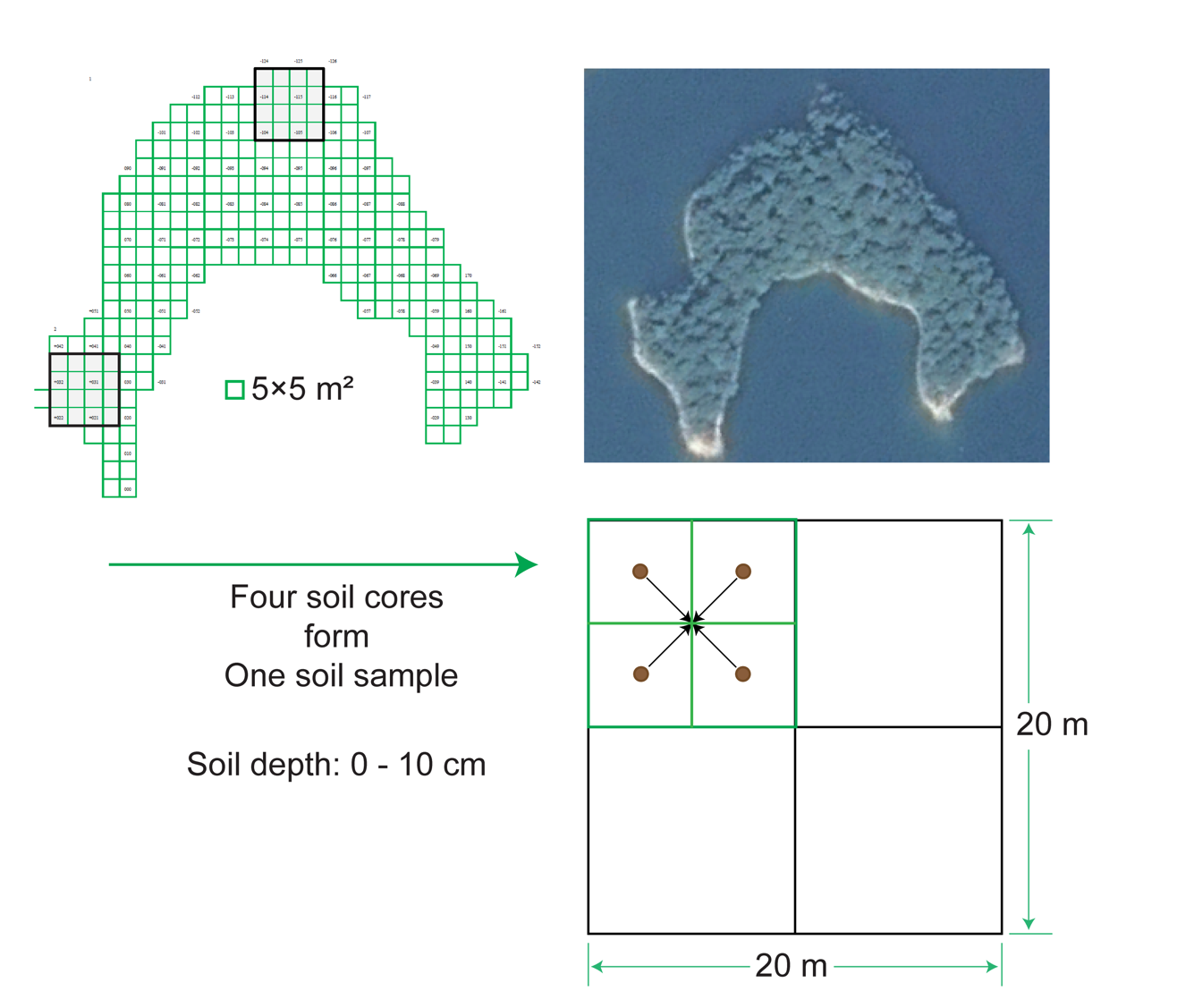


**Fig. S10** **Schematic diagram of soil sampling in one island.**


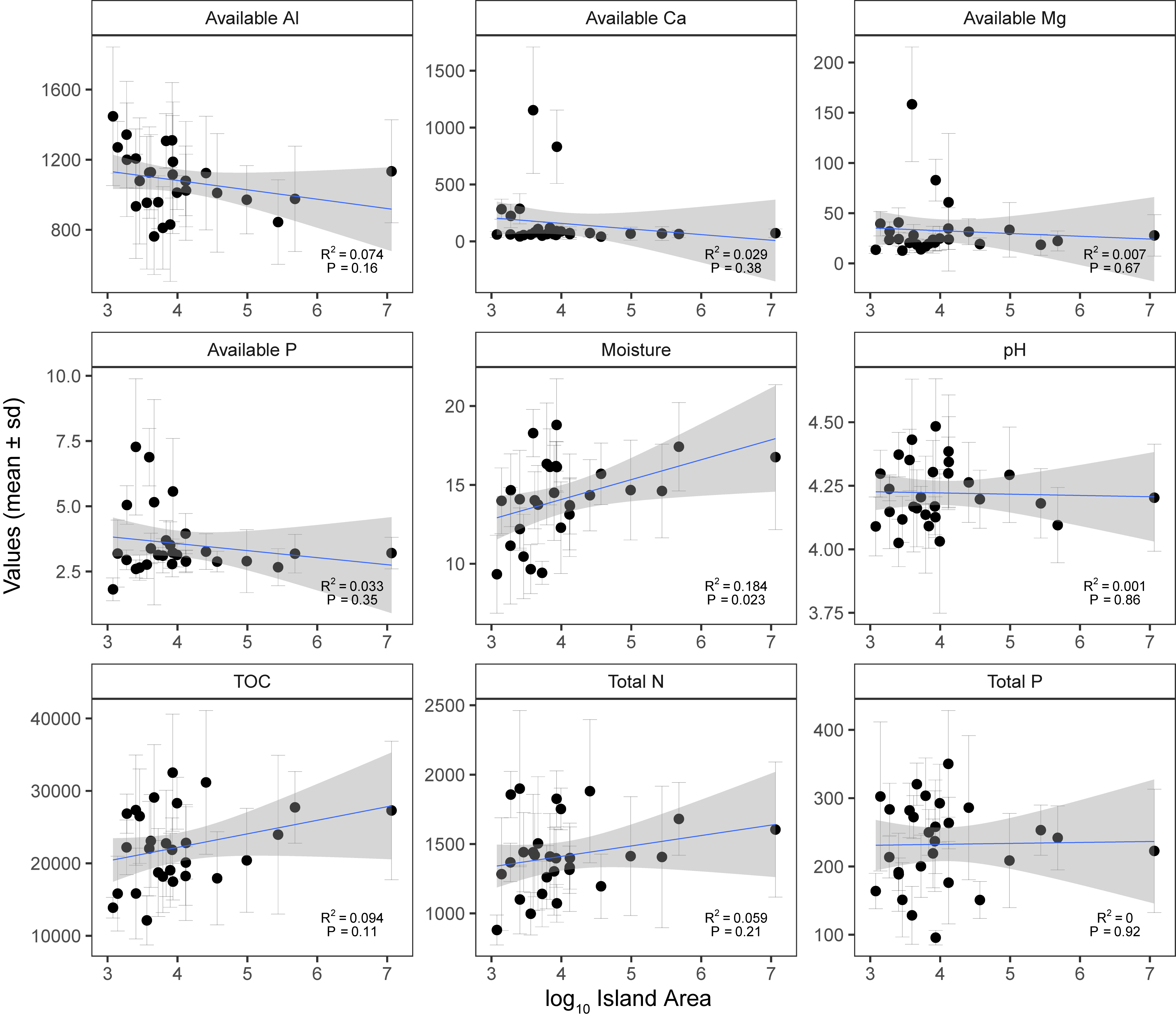


**Fig. S11** **The relationship between island area (m^2^ ; log10 -transformed) and each soil property (mean ± sd).** The unit of moisture is %; the unit of all other soil properties is ppm. Grey shadows represent 95% confidence interval.


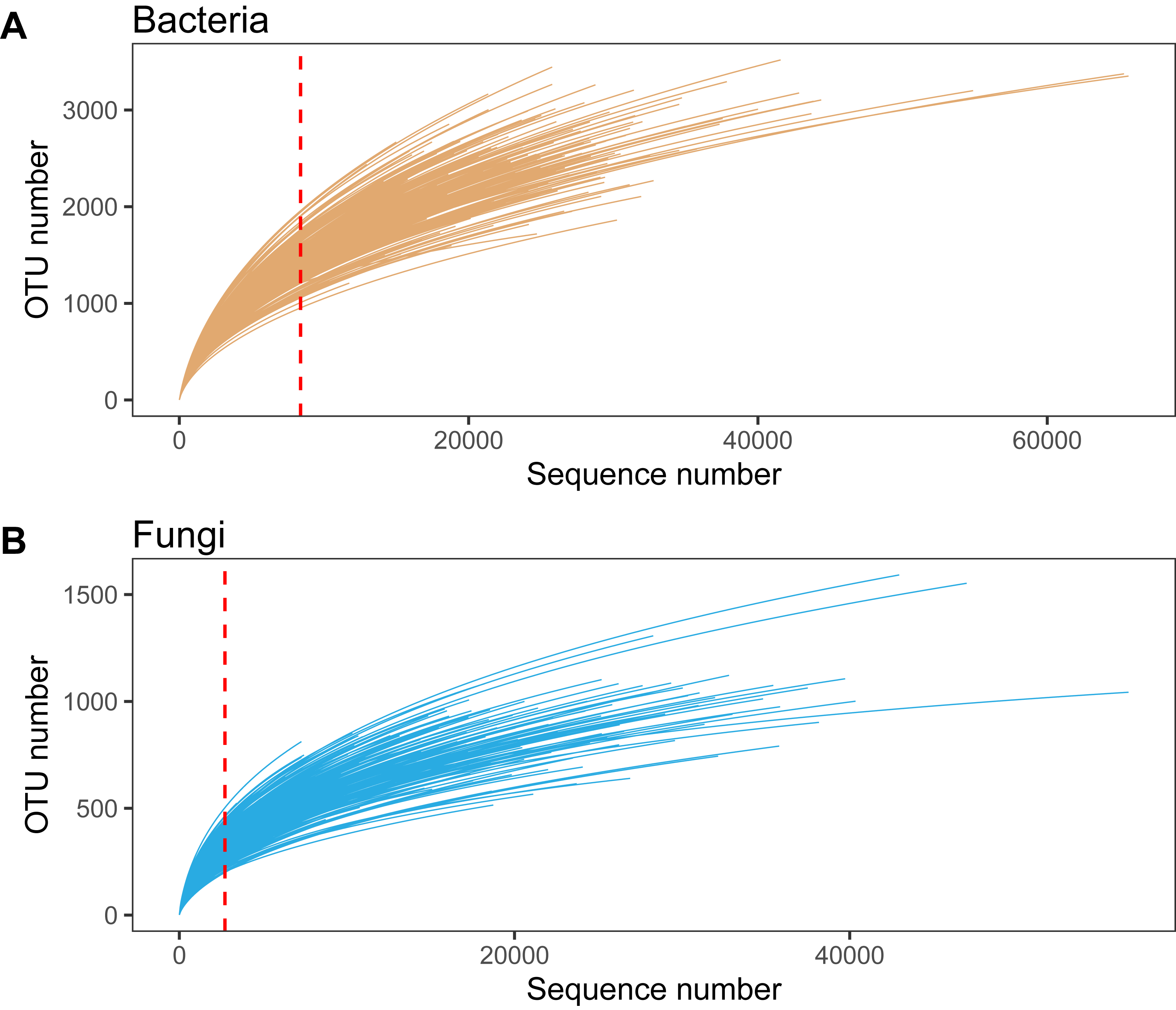


**Fig. S12 Rarefaction curves of bacterial and fungal communities for each sample.** The vertical red dash lines indicate the minimum sequence numbers that were used to for rarefying bacterial (**A**) and fungal (**B**) community of each sample, respectively.
