# Supplementary material for: Spatial scaling of soil microbial co‐occurrence networks in a fragmented landscape
Source: mLife. 2023 Jun 26;2(2):209–15. doi: 10.1002/mlf2.12073 (PMC10989968; doi:10.1002/mlf2.12073)
Supplement: Supplementary file 3 — Supplementary information. [file MLF2-2-209-s001.docx]

# *Supporting information for*

# Spatial scaling of soil microbial co-occurrence networks in a fragmented landscape

Pandeng Wang^1,2^, Shao-Peng Li^3,4,#^, Xian Yang^1,2^, Xingfeng Si^3,4^, Wen-Jun Li^1^, Wensheng Shu^5^, Lin Jiang^2^

^1^ State Key Laboratory of Biocontrol, School of Ecology & School of Life Sciences, Sun Yat-Sen University, Guangzhou 510275, China

^2^ School of Biological Sciences, Georgia Institute of Technology, Atlanta, GA 30332, USA

^3^ Zhejiang Tiantong Forest Ecosystem National Observation and Research Station, School of Ecological and Environmental Sciences, East China Normal University, Shanghai 200241, China

^4^ Institute of Eco-Chongming (IEC), Shanghai 200062, China

^5^ School of Life Sciences, South China Normal University, Guangzhou 510631, China

***# Author for correspondence:***

Shao-Peng Li (Tel: 86(021)54341138; Fax: 86(021)54341138; Email: spli@des.ecnu.edu.cn)

# Materials and methods

*Study sites and soil sampling*

We selected 29 islands from the Thousand Island Lake (29°22′N to 29°50′N and 118°34′E to 119°15′E) in subtropical China as our study sites (Fig. S9). These islands were formed by damming in 1959, which led to the elimination of almost all hardwood plants. Currently, after more than 60 years of natural succession, these islands are dominated by a single pine species (*Pinus massoniana*). As larger islands are likely to harbor more species and require more sampling efforts to achieve comparable coverage to smaller islands [^1^](#_ENREF_1), we implemented proportional sampling to achieve a fair comparison between islands of different sizes. Within each island, 1 to 6 permanent 20 × 20 m^2^ plots were established, with the number of plots roughly proportional to the island area on a logarithmic scale (Table S5). The smallest island, where we were unable to establish one permanent plot, was excluded from our analysis. Each plot was further divided into four quadrats (10 × 10 m^2^). In mid-May 2015, we collected four soil cores from the four corners of each quadrat and mixed them evenly to form one composite sample (Fig. S10), therefore four samples were collected for each plot. In total, we obtained 284 samples from the 28 islands. Detailed sampling regimes were previously described [^2^](#_ENREF_2)^,^ [^3^](#_ENREF_3).

*Soil properties, DNA extraction and sequencing*

We measured nine soil chemistry variables, including pH, soil moisture, total organic carbon (TOC), total P and N, and available P, Ca, Al, Mg (Fig. S11). The detailed procedures have been described in a previous study [^2^](#_ENREF_2). In brief, soil moisture was measured by drying (105 °C) fresh soil to constant weight; pH was determined by pH meter with soil suspension (soil:water, 1:2.5); TOC and total N were measured by the potassium dichromate oxidation method and the Semimicro-Kjeldahl method, respectively; total P and available P content were measured using the colorimetric method; available Ca, Al, and Mg contents were measured by Inductively Coupled Plasma Optical Emission Spectrometry (ICP-OES, Optima 2100DV; Perkin-Elmer, Massachusetts, USA).

We extracted DNA for each soil sample using the MoBio PowerSoil DNA extraction kit (Qiagen, Carlsbad, CA, USA). Then, we amplified the V4 region of 16S rRNA gene with the primer set 515 (GTGCCAGCMGCCGCGGTAA) and 806 (GGACTACHVGGGTWTCTAAT) to profile bacterial communities, and ITS2 region with the primer set ITS3 (GCATCGATGAAGAACGCAGC) and ITS4 (TCCTCCGCTTATTGATATGC) to profile fungal communities. Detailed procedures were previously described [^2^](#_ENREF_2). PCR products from all samples were pooled in equimolar concentrations and purified using the E.Z.N.A.^®^ Gel Extraction Kit (Omega BioTek, Doraville, USA). DNA sequencing was performed on a 2×300 bp paired-end Illumina MiSeq platform (Illumina; San Diego, CA, USA). Raw sequence data have been deposited to NCBI under the project number PRJNA517449.

*Bioinformatic analysis*

We processed the sequencing data following the MOTHUR and USEARCH pipelines [^4^](#_ENREF_4)^,^ [^5^](#_ENREF_5). After assembling paired reads (mismatches < 5%), trimming barcodes and primers, and filtering low quality reads (pdiffs=2, bdiffs=0, maxambig=0, maxhomop=12, qwindowsize=50, qwindowaverage=25, minlength=200) by using MOTHUR v1.36.1, we used USEARCH v8 to dereplicate and cluster high-quality sequences into operational taxonomic units (OTUs) at the 97% similarity threshold and removed chimeras using the UPARSE algorithm [^5^](#_ENREF_5). OTU table was generated by mapping high-quality reads against OTUs’ representative sequences. After removing singletons, the taxonomy of each OTU was determined by using the Ribosomal Database Project (RDP) classifier [^6^](#_ENREF_6) against the SILVA v123 database [^7^](#_ENREF_7) for bacteria and the UNITE v7.0 database [^8^](#_ENREF_8) for fungi. OTUs that were not classified into bacteria and fungi were removed before subsequent analyses. Each sample was rarefied to equal sequencing depth (8381 and 2720 for bacteria and fungi, respectively) to correct sampling effects (Fig. S12).

*Network analyses*

We constructed soil bacterial and fungal co-occurrence networks following the MENA pipeline (<http://ieg4.rccc.ou.edu/mena>) using all 284 samples. First, to mitigate unreliable network inference caused by rare species, we only kept OTUs that occurred in at least 12 samples, as rare OTUs may not provide sufficient abundance information for accurate correlation inferences. We then generated the co-occurrence networks of soil bacteria and fungi based on the Spearman correlations of OTU abundances (log-transformed) across all 284 samples. The correlation cut-off thresholds were determined by an RMT-based approach [^9^](#_ENREF_9), which automatically identifies the appropriate thresholds according to the predictions of random matrix theory. We obtained the sub-network of each island based on the occurred OTUs in each island using the *igraph* package [^10^](#_ENREF_10). The network properties, including *n* (network size), *L* (total links), average K (average degree), and connentance, were also calculated by the R package *igraph*. Following previous work [^11^](#_ENREF_11), we calculated RM (relative modularity = ${(M-\bar{M_{r}})}/{\bar{M_{r}}}$; *M*, modularity of the observed network, $\bar{M_{r}}$, mean modularity of the random networks) to quantify the degree to which a network is compartmentalized into different modules. RM, which removes the effects of network size and total links, is more suitable than absolute modularity for comparison across islands. Additionally, we also identified potential keystone species for each island based on the nodes’ within-module connectivity (Z_i_) and among-module connectivity (P_i_) [^12^](#_ENREF_12). Following previous wrok [^9^](#_ENREF_9)^,^ [^11^](#_ENREF_11)^,^ [^13^](#_ENREF_13), nodes in a network were classified into network hubs (Z_i_ ≥ 2.5, P_i_ ≥ 0.62), module hubs (Z_i_ ≥ 2.5, P_i_ < 0.62), connectors (Z_i_ < 2.5, P_i_ ≥ 0.62), and peripherals (Z_i_ < 2.5, P_i_ < 0.62). We classified network hubs, module hubs, and connectors as potential keystone taxa in this study.

To measure network stability, most previous theoretical studies focus on the internal (local) stability of community that is in an equilibrium state via examining the properties of the Jacobian matrix [^14^](#_ENREF_14). However, the majority of natural ecological communities are not in equilibrium, and it is difficult to derive the elements of the Jacobian matrix from field surveys. Thus, empirical studies have often focused on the external stability (resistance to extinction) of natural communities [^15^](#_ENREF_15). Robustness and vulnerability are two indexes measuring the external stability and instability of ecological networks, respectively. Robustness quantifies the resistance of ecological network to species extinction [^16^](#_ENREF_16), whereas vulnerability measures the efficiency of the spread of local perturbations across the network (i.e., how fast the effects of environmental changes on impacted nodes transmit to other network components) [^11^](#_ENREF_11). Therefore, network stability increases with its robustness, but decreases with its vulnerability. Here, we calculated network robustness and vulnerability following Yuan et al. [^11^](#_ENREF_11). Robustness was calculated as the proportion of the remaining nodes in the network after randomly removing 50% nodes. Network vulnerability was calculated as the maximal vulnerability of nodes in the network. The vulnerability of each node was measured as ${(E-E_{i})}/E$ (E represents the network global efficiency, E*_i_* represents the network global efficiency after removing node *i* and all its links).

To examine the potential effects of indirect associations on network properties and stability, we used a state-of-the-art framework, named iDIRECT [^17^](#_ENREF_17) (Inference of Direct and Indirect Relationships with Effective Copula-based Transitivity), to disentangle the direct and indirect associations in the soil bacterial and fungal networks. After removing the indirect associations from the whole networks, we performed the same network analyses as aforementioned basing on the iDIRECT-processed networks.

*Statistical analyses*

To explore NARs, we conducted liner regressions of network properties (including *n*, *L*, average K, connentance, RM, and number of keystones) and stability (robustness and vulnerability) against island area (m^2^; log_10_-transformed). To determine the drivers of the variations in soil bacterial and fungal network properties, we performed random forest analysis with bacterial/fungal richness and soil properties (including pH, soil moisture, TOC, total P and N, and available P, Ca, Al, Mg) as explanatory variables. Explanatory variables that could not increase the model predictive ability were excluded from the final random forest model. We further calculated Spearman correlations between network properties and network stability to assess their relationships. To determine the potential roles of keystone taxa in maintaining network stability, we also calculated the robustness of keystone-removed networks and compared to the original networks using *t*-test.

# References

1. Schoereder JH, Galbiati C, Ribas CR, Sobrinho TG, Sperber CF, DeSouza O, et al. Should we use proportional sampling for species–area studies? *J Biogeogr*. 2004;31:1219-1226.

2. Li SP, Wang PD, Chen YJ, Wilson MC, Yang X, Ma C, et al. Island biogeography of soil bacteria and fungi: similar patterns, but different mechanisms. *ISME J*. 2020;14:1886-1896.

3. Wang PD, Li SP, Yang X, Zhou JZ, Shu WS, Jiang L. Mechanisms of soil bacterial and fungal community assembly differ among and within islands. *Environ Microbiol*. 2020;22:1559-1571.

4. Schloss PD, Westcott SL, Ryabin T, Hall JR, Hartmann M, Hollister EB, et al. Introducing mothur: Open-Source, Platform-Independent, Community-Supported Software for Describing and Comparing Microbial Communities. *Appl Environ Microbiol*. 2009;75:7537-7541.

5. Edgar RC. UPARSE: highly accurate OTU sequences from microbial amplicon reads. *Nat Methods*. 2013;10:996-998.

6. Wang Q, Garrity GM, Tiedje JM, Cole JR. Naive Bayesian classifier for rapid assignment of rRNA sequences into the new bacterial taxonomy. *Appl Environ Microbiol*. 2007;73:5261-5267.

7. Quast C, Pruesse E, Yilmaz P, Gerken J, Schweer T, Yarza P, et al. The SILVA ribosomal RNA gene database project: improved data processing and web-based tools. *Nucleic Acids Res*. 2012;41:D590-D596.

8. Abarenkov K, Henrik Nilsson R, Larsson KH, Alexander IJ, Eberhardt U, Erland S, et al. The UNITE database for molecular identification of fungi–recent updates and future perspectives. *New Phytol*. 2010;186:281-285.

9. Deng Y, Jiang Y-H, Yang Y, He Z, Luo F, Zhou J. Molecular ecological network analyses. *BMC Bioinform*. 2012;13:113.

10. Csardi G, Nepusz T. The igraph software package for complex network research. *Int J Complex Syst*. 2006;1695:1-9.

11. Yuan MM, Guo X, Wu L, Zhang Y, Xiao N, Ning D, et al. Climate warming enhances microbial network complexity and stability. *Nat Clim Chang*. 2021;11:343-348.

12. Guimera R, Nunes Amaral LA. Functional cartography of complex metabolic networks. *Nature*. 2005;433:895-900.

13. Olesen JM, Bascompte J, Dupont YL, Jordano P. The modularity of pollination networks. *Proc Natl Acad Sci USA*. 2007;104:19891-19896.

14. Allesina S, Tang S. The stability–complexity relationship at age 40: a random matrix perspective. *Popul Ecol*. 2015;57:63-75.

15. Jacquet C, Moritz C, Morissette L, Legagneux P, Massol F, Archambault P, et al. No complexity–stability relationship in empirical ecosystems. *Nat Commum*. 2016;7:12573.

16. Montesinos-Navarro A, Hiraldo F, Tella JL, Blanco G. Network structure embracing mutualism–antagonism continuums increases community robustness. *Nat Ecol Evol*. 2017;1:1661-1669.

17. Xiao N, Zhou A, Kempher ML, Zhou BY, Shi ZJ, Yuan M, et al. Disentangling direct from indirect relationships in association networks. *Proc Natl Acad Sci USA*. 2022;119:e2109995119.
